# Supplementary material for: Ion-beam assisted laser fabrication of sensing plasmonic nanostructures
Source: Sci Rep. 2016 Jan 18;6:19410. doi: 10.1038/srep19410 (PMC4726055; doi:10.1038/srep19410)
Supplement: Supplementary Information [file srep19410-s1.doc]

Supplementary Information

Ion-beam assisted laser fabrication of sensing plasmonic nanostructures

Aleksandr Kuchmizhak,^*^ Stanislav Gurbatov, Oleg Vitrik, Yuri Kulchin, Valentin Milichko, Sergey Makarov, and Sergey Kudryashov

**
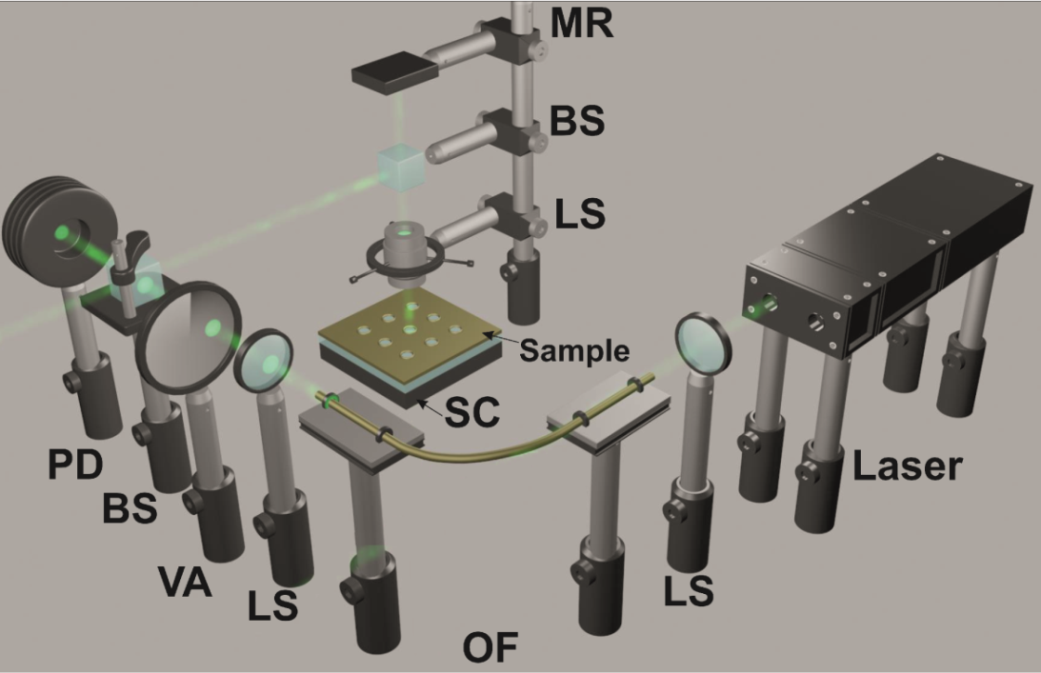
**

**Fig.S1. Schematic of the experimental setup:** LS- lens, MR – mirror, BS – beam splitter, PD – photodetector, OF – optical fiber, SC – scanning platform, VA – variable energy attenuator.

**
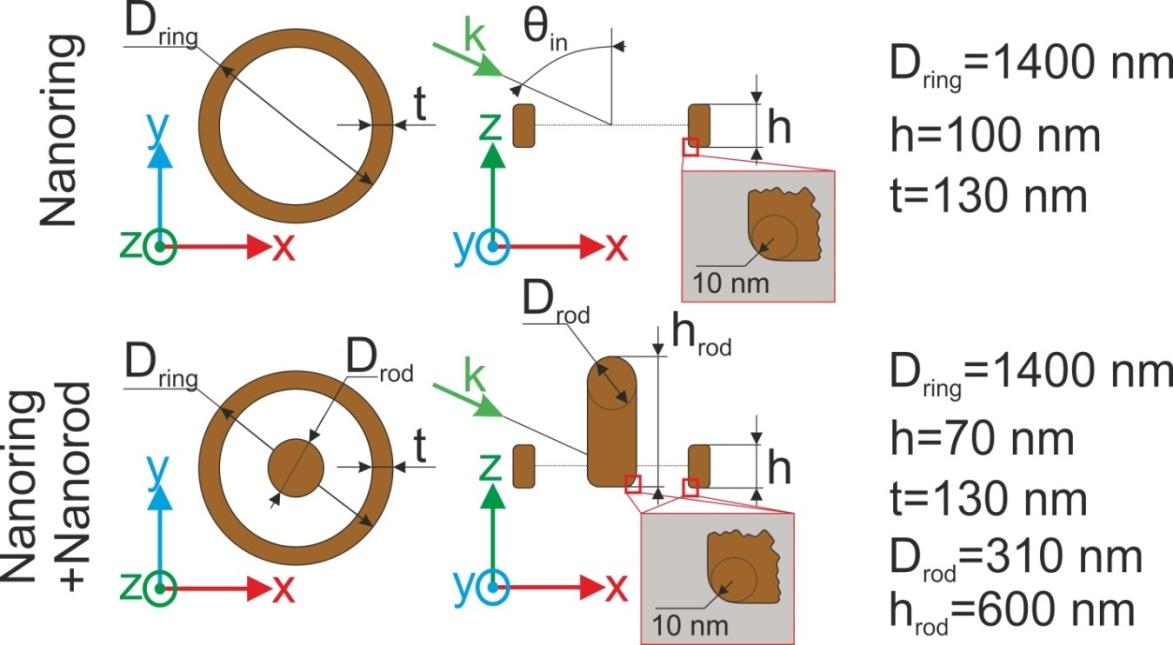
**

**Fig.S2.** **The details of the FDTD simulations of the nanorings and nanoring-nanorod ensembles.**

**
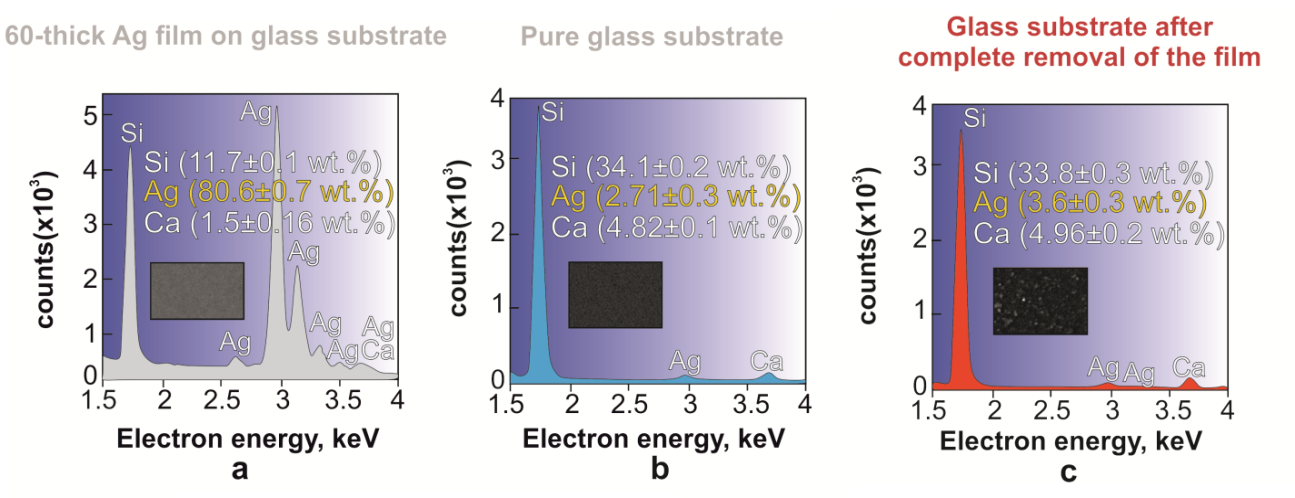
**

**Fig.S3. EDX spectra** measured for the 60-nm-thick Ag film on the glass substrate (a), the pure glass substrate (b) and for the glass substrate after complete removal of the Ag film (c). Insets show SEM images of the corresponding areas (5-μm wide), where the data acquisition was performed.

**Supplementary note.**

To ensure that the entire metal film was completely removed from the glass substrate after Ar^+^-beam polishing procedure, we have acquired and compared EDX spectra from the 60-nm-thick Ag film on the glass substrate, pure glass substrate as well as from the polished glass surface near the fabricated plasmonic nanostructures (Fig.S3). As seen, the relative Ag-content from the polished surface is slightly (less than 1 wt.%) higher, than the reference noise level measured for the pure glass substrate, and is significantly smaller, than the Ag-content for the metal film, indicating that very small amount of silver remained on/inside the glass substrate. We believe that the residual separate small Ag nanocrystallites won’t form a continuous film, thus not influencing significantly on the properties of the fabricated isolated functional plasmonic nanostructures. Note that, EDX spectra acquired for the Au and Cu nanostructures, demonstrated similar values of the corresponding metal content on the polished glass substrates after the complete removal of these metal films.
